# Conservation genetics and evolution in an endangered species: research in Sonoran topminnows*

**DOI:** 10.1111/j.1752-4571.2012.00259.x

**Published:** 2012-12

**Authors:** Philip W Hedrick, Carla R Hurt

**Affiliations:** 1 School of Life Sciences, Arizona State UniversityTempe, AZ, USA; 2 Department of Biology, University of MiamiCoral Gables, FL, USA

**Keywords:** evolutionary significant units, inbreeding depression, major histocompatibility complex, mtDNA, outbreeding depression, reproductive isolation

## Abstract

Conservation genetics of endangered species has primarily focused on using neutral markers to determine units of conservation and estimating evolutionary parameters. Because the endangered Sonoran topminnow can be bred in the laboratory and has a relatively short generation length, experiments to examine both detrimental and adaptive variations are also possible. Here, we discuss over two decades of empirical and experimental observations in the Sonoran topminnow. Results from this research have been used to determine species and evolutionary significant units using neutral markers, document inbreeding and outbreeding depression and genetic load using experimental crosses, and measure adaptive differences in fitness-related traits and variation in pathogen resistance among populations and major histocompatibility complex genotypes. In addition, both premating and postmating reproductive isolation between Gila and Yaqui topminnows have been experimentally determined, and the predicted and observed ancestry of these two species in experimental crosses has been examined over time. Although some have suggested that endangered species are unsuitable for experimentation because of both practical and ethical considerations, these results demonstrate that in this case an endangered species can be employed to examine fundamental questions in conservation and evolution.

## Introduction

For the past half century, it has been widely recognized that the rate of species extinction was increasing and that many other species were in imminent extinction danger. The major factors related to these extinctions and declines were overharvesting from hunting, fishing, trapping, and other killing; loss, degradation, and fragmentation of habitat; and introduction of non-native species such as pathogens, parasites, predators, and competitors (Diamond 1989). Minckley and Deacon (1991) detailed the effects of many of these factors on the native fishes of the arid western United States in the same period (see also Minckley and Marsh 2009). Because of the limited geographic distribution for many aquatic species in arid lands, they are particularly susceptible to habitat degradation and fragmentation, introduced non-natives species, and other factors potentially causing population declines and extinction.

Conservation biology was developed to understand the processes influencing extinction. Genetics has been an important focus of conservation biology because it helps determine the evolutionary context of endangered species and enables the development of better management strategies. Genetic variation interpreted in a population genetics context can be used to reconstruct the evolutionary history, examine the contemporary status, and predict the future of endangered species. Overall, the framework of evolutionary genetics theory furnishes an elegant approach to interpret the measured amounts of genetic variation and predict the future effects of evolutionary factors and management strategies.

Model organisms have sometimes been used to examine the bases of conservation management approaches. In particular, some experiments using the fruitfly *Drosophila* have compared different management options; however, these experiments appear to be of rather limited use in vertebrate conservation genetics. Although such fruitfly laboratory experiments may serve a useful heuristic purpose to illustrate evolutionary genetics principles or management options, it seems unlikely that laboratory experiments on insects with a history of a very large population size will provide new insight into conservation of endangered species, most of which are vertebrates with small population size, have a history of declining numbers, might have important social and mating structures, and so on. Furthermore, if laboratory experiments on a model organism give counterintuitive findings, those results are probably only relevant to the model organism that is being used and not for endangered species in general.

Some have suggested that endangered species themselves are unsuitable for experimentation because of both practical and ethical considerations. However, experimentation has been possible on several endangered species, for example the Gila and Yaqui topminnows discussed below, Pacific salmon (Arkush et al. 2002), and a few other species which have given insights into conservation that would have otherwise been impossible. In addition, individuals of many endangered species are often monitored with great specificity, providing more detailed data than are available for nonendangered species (Vilà et al. 2003; Johnson et al. 2010; Adams et al. 2011). This tracking allows an understanding of details about movement, mating, life history, etc. of endangered species not generally possible from laboratory experiments, theory, or less-intensive studies.

Below, we will present a review of the extensive research on two species of the endangered Sonoran topminnow, the Gila and the Yaqui topminnow, most of which was conducted by us and our colleagues. Our research includes both empirical observation and laboratory experimentation, which we were able to do because we had permission to collect small numbers of fish, bring them into the laboratory, increase their numbers, and provide refugia for them. We were then allowed to carry out experiments on excess fish following approved protocols. Although we do not consider these fish as a model organism, they do have some attributes of one, that is, good survival and reproduction under appropriate laboratory conditions, short generation length, small size, etc. However, we feel that extreme care should be taken when generalizing our results from topminnows to other endangered species.

As an organizational theme for our evaluation of evolutionary and conservation genetics in Gila and Yaqui topminnows, three major types of genetic variation – neutral, detrimental, and adaptive – can be used (Hedrick 2001). Of course, whether particular variation is neutral, detrimental, or adaptive depends upon the environment, population size, genetic background, etc. For example, a particular allele that is adaptive, say provides resistance to an infectious disease in one environment, may be detrimental when the pathogen is absent because of a pleiotropic cost associated with the allele. Likewise, genetic variants that are neutral in one situation may be adaptive in another. In addition, we will discuss genetics related to reproductive isolation and speciation in these two species of Sonoran topminnows. From this perspective, it seems that not only genetic research has contributed substantially to the conservation of these species but research in these endangered species has also contributed to general knowledge in evolutionary biology.

**Figure 1:**
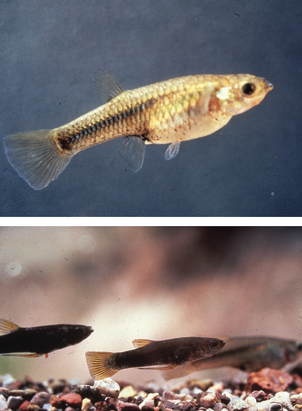
Photographs of Gila topminnows with top photograph showing a female and the lower photograph showing two males in dark mating color and a female behind them. Notice the male sexual organ, the gonopodium, in the two males (photograph courtesy of J. Rinne).

As background, the Sonoran topminnow is a small (<50 mm) live-bearing fish of the family Poeciliidae that occurs in Arizona, United States, and Sonora, Mexico (Fig. 1). There are two federally listed endangered Sonoran topminnow taxa, the Gila topminnow (*Poeciliopsis occidentalis*) and the Yaqui topminnow (*Poeciliopsis sonoriensis*), which are morphologically nearly identical. They have allopatric distributions: the Gila topminnow occurs in the Gila River basin that starts in New Mexico and flows west into the Colorado River and the Yaqui topminnow occurs in the Yaqui River drainage that flows south from southeastern Arizona across Mexico into the Sea of Cortez.

**Figure 2:**
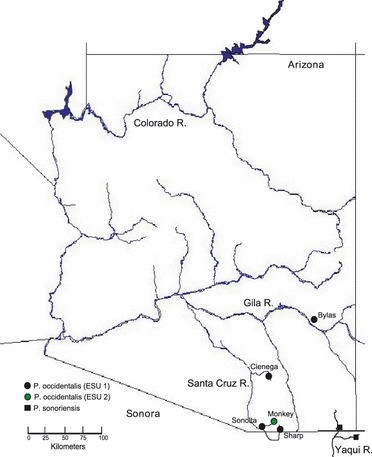
The locations of the natural populations of the Gila and Yaqui topminnows in the USA. There are actually two populations of Gila topminnows at Bylas and four populations at Sonoita Creek, Cottonwood Spring is near Monkey Spring, and the population of Yaqui topminnows at Cajon Benito, Sonora, is indicated.

The Gila topminnow was once considered among the most abundant fishes in the lower Gila River basin in Arizona. They now persist in only a few watersheds in southeastern Arizona (Fig. 2), primarily because of loss and fragmentation of adequate shallow-water habitat and the widespread introduction of another livebearer, the non-native western mosquitofish (*Gambusia affinis*) (Minckley 1999). The Yaqui topminnow was never widespread in the United States because the Yaqui River drainage includes only a small part of extreme southeastern Arizona, now within the San Bernadino National Wildlife Refuge (Fig. 2). It has faced similar threats to its persistence. Below, we will focus on populations of both taxa in the United States because there is little known about conservation genetics of these species in Mexico. For a summary of the earlier genetic research on these taxa and other related ones, see Vrijenhoek (1996, 1998).

## Neutral variation

The extent and pattern of molecular variation within a population is generally consistent with neutrality, that is, a balance predicted by a reduction in variation from genetic drift and an increase in variation from mutation. Even if selection is acting on the variation at a given gene, when the population is small, genetic drift may have a greater effect than selection. In general, neutrality of genetic variants can be assumed when the selection coefficient *s* is <1/(2*N*_*e*_) where *N*_*e*_ is the effective population size (Kimura 1983). In other words, because of the generally low effective population size in endangered species, genetic variants are more likely to be effectively neutral in endangered than in common species. As a result, in endangered species, there may be both effectively less selection acting to maintain favorable genetic variation and to eliminate detrimental variation.

The application of neutral genetic variation is widespread in conservation genetics and has been used in various ways for identification and estimation in conservation genetics. For example, neutral genetic variation has been used to identify individuals, parents of particular individuals, populations, management units (MUs), evolutionary significant units (ESUs), and species. As background, MUs have been defined as ‘populations that have diverged in allelic frequency and that are significant for conservation in that they represent populations connected by such low levels of gene flow that they are functionally independent’ and ESUs as groups that show phylogeographic differentiation for mtDNA variants and significant divergence of allele frequencies at nuclear loci (Moritz 1994, 1999; however, see Waples 1995; Crandall et al. 2000; Fraser and Bernatchez 2001). By appropriately identifying MUs and ESUs, conservation of endangered species should allow management actions to be more effective. In Sonoran topminnows, neutral variation has been used in the identification of species, ESUs, and MUs, and we will review some of these findings below. In addition, neutral genetic variation has been used to estimate various evolutionary and genetic parameters important in conservation genetics, such as effective population size, genetic bottlenecks, gene flow, population structure, relationships between groups, individual inbreeding coefficients, and female and male ancestry.

### Species

Controversy over the species status of Sonoran topminnows has resulted in several taxonomic changes since its initial description as two distinct species, *P. occidentalis* (Gila topminnow) and *P. sonoriensis* (Yaqui topminnow) (Girard 1859). Several authors either synonymized or retained these taxa (for a review, see Quattro et al. 1996). Based on subtle morphological differences, Minckley (1969, 1971) redescribed the two taxa as different subspecies, *P. o. occidentalis* (Gila topminnow) and *P. o. sonoriensis* (Yaqui topminnow). Since being recognized as endangered, Sonoran topminnows have been surveyed for a variety of molecular markers in an effort to understand their population structure within and differentiation between taxa. Based on this genetic evidence and other considerations, Minckley (1999) suggested that the two taxa should be again considered different species, *P. occidentalis* (Gila topminnow) and *P. sonoriensis* (Yaqui topminnow).

Collectively, these molecular genetic studies suggest that *P. occidentalis* and *P. sonoriensis*, although morphologically very similar, have long been isolated. Quattro et al. (1996) examined mtDNA variation and found that Gila and Yaqui topminnows were fixed for two very divergent haplotypes. Mateos et al. (2002) examined cytochrome B and ND2 in two Gila and two Yaqui topminnows from Mexico and one Yaqui topminnow from Arizona and found that all the Gila and Yaqui topminnow sequences were substantially different. Subsequently, sequences of ND2, cytochrome B, and the D loop (2626 base pairs total) were found to be invariant in large samples within the two taxa in the United States but were quite divergent between the two taxa. Overall, the species differed by 29 mtDNA nucleotides, and they were 1.1% divergent, suggesting that the two species had been separated for approximately one million years (Hedrick et al. 2006). Interestingly, based on a survey of allozyme loci, Vrijenhoek et al. (1985) suggested that Gila and Yaqui topminnows had diverged about 1.7 million years ago. However, they found substantial divergence between some Gila topminnow populations and great similarity between Gila and Yaqui topminnow populations (Meffe and Vrijenhoek 1988), so they continued to identify Gila and Yaqui topminnows as one species.

Hedrick et al. (2001a) also examined variation at ten microsatellite loci. For the seven microsatellite loci that could be scored in both taxa, the alleles formed nearly nonoverlapping (diagnostic) sets of alleles at these loci (Table 1). A total of 39 and 22 alleles were identified in the Gila and Yaqui topminnows, respectively; only two of these were shared in the two taxa. Overall, the frequency of Yaqui alleles in Gila samples was only 1.2%, and the frequency of Gila alleles in Yaqui samples was only 1.1%.

**Table 1:** Allele frequencies for seven microsatellite loci in Gila and Yaqui topminnows with their allele sizes in base pairs given in the allele designations. Where a range of allele size is given, two or more alleles were found in that size range. The average gives the frequency of diagnostic Gila and Yaqui alleles in Gila and Yaqui topminnows (Hedrick et al. 2001a)

	Species		
	
Locus	Alleles	Gila	Yaqui
G-49	149–159	0.917	–
	161	0.083	0.486
	163	–	0.514
C-15	164–192	–	1.0
	202–248	1.0	–
OO56	143–153	1.0	–
	256	–	1.0
LL53	110–116	–	1.0
	136–164	1.0	–
4-44	106	–	0.526
	108	1.0	0.080
	114, 118	–	0.394
Acc	124	–	0.794
	128	1.0	–
	130	–	0.206
G53	96, 102, 104	–	1.0
	100	1.0	–
Average	Gila alleles	0.988	0.011
	Yaqui alleles	0.012	0.989

In addition, for three microsatellite loci, the sizes of the alleles in the two species were greatly divergent (Table 1). The largest difference was for locus *OO56*, which differed by more than 100 base pairs between the species. However, this locus is a complicated repeat (Parker et al. 1998), so it is not clear how many mutational steps are responsible for the difference in size between species. The other two loci that have disjunct size distributions, *C-15* and *LL53*, are simple GT dinucleotide repeats. Because the most common mutation at perfect repeat microsatellite loci is thought to differ by only a single repeat, it appears that isolation between these taxa must been long and complete for so many differences to accumulate.

In addition, Hedrick et al. (2001a) found extensive variation at an major histocompatibility complex (MHC) locus, which we will discuss below in the section on adaptive variation, within both Gila (17 alleles) and Yaqui topminnows (12 alleles). These 29 alleles comprised two nonoverlapping sets in the two taxa such that the alleles can be used as diagnostic markers for the two taxa.

Overall, the molecular genetic data from mtDNA sequences, microsatellite loci, and MHC sequences strongly support extensive divergence and long-term isolation of Gila and Yaqui topminnows in spite of their morphological similarity. In addition, geological evidence suggests that the Gila and Yaqui drainages were separated during the late Pliocene or early Pleistocene (Melton 1960; Minckley et al. 1986). Below, we will discuss our experimental research that demonstrates incipient reproductive isolation between these allopatric taxa.

### ESUs

**Figure 3:**
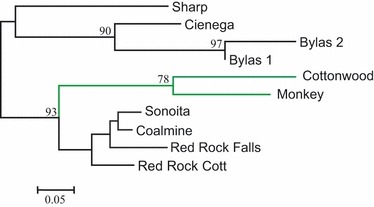
An unrooted neighbor-joining tree for the 10 natural populations of Gila topminnows based on the allele frequencies at microsatellite loci and an major histocompatibility complex locus. Note that at the locations indicated in Fig. 2, there are two populations of Gila topminnows very close together at Bylas (Bylas 1 and 2) and four populations very close in Sonoita Creek (Sonoita Creek, Coalmine Canyon, Red Rock Falls, and Red Rock Cott Tank), and Cottonwood Spring is very near Monkey Spring (for a more detailed map, see Hedrick et al. 2001a). The numbers indicate bootstrap values for the nodes in the tree from Hedrick et al. (2001a), and the Monkey–Cottonwood Spring evolutionary significant unit is indicated in green.

Often, genetic variants are used in endangered species to identify ESUs and management units (MUs) (Moritz 1994, 1999). Hedrick et al. (2001a) surveyed the 10 known natural populations of Gila topminnows for variation at microsatellite loci and an MHC locus to determine the population structure in this species. Based on the data from these loci, the natural populations appear to fall into five groups that Hedrick et al. (2001a) placed into two ESUs (Fig. 3). They suggested that one of these ESUs, which contained eight of the natural populations (excluding Monkey and Cottonwood Springs), contained four MUs. Other distributional and ecological attributes of these populations support these groupings (Parker et al. 1999; Hedrick et al. 2001a).

Hedrick et al. (2001a) suggested that the other ESU includes the Monkey and Cottonwood Springs populations that are nearby (five stream km apart) in the upper Sonoita Creek watershed. These two populations are genetically similar to each other and have intermediate levels of genetic variation. Parker et al. (1999) found Monkey Spring to be the genetically most differentiated population and the distinctiveness of this population is further supported by geological, ecological, and life history evidence. Monkey Spring has been isolated from Sonoita Creek, into which it flows, through formation of a travertine dam, for perhaps 10 000 years. It is the only population inhabiting a warm spring site (28°C) and does not have the extreme seasonal temperature variation experienced by the other populations.

As indicators of the long-term isolation of this site from immigration, it was once occupied by a now-extinct species of pupfish and a now-extinct, morphologically distinct, form of the Gila chub. Also, the springsnail (*Pyrgolopsis thompsoni*) found in Monkey Spring has a very divergent mtDNA sequence from other *P. thompsoni* populations (Hurt 2004). In addition, this population has a substantial life history difference, in that male development time was 50% longer in Monkey Spring males than from other sites (Cardwell et al. 1998), and upon inbreeding, it produces virtually all females (Sheffer et al. 1999). The Gila topminnows are still present in both Monkey and Cottonwood Springs and are in captivity at several locations.

As for the four MUs in the other ESU (R. Timmons, personal communication), Gila topminnows in Sharp Spring have gone extinct, apparently because of mosquitofish, but the Sharp Spring MU has been maintained in several populations at other sites and several captive populations. The Cienega Creek MU is still extant and has naturally expanded (and is in captive populations). The Bylas Spring MU now consists of three natural populations, several populations at other sites, and several captive populations. Finally, in the Sonoita Creek MU, the natural Red Rock populations have gone extinct (because of drought and mosquitofish) but are still in captive populations, while the lower Sonoita Creek populations, Sonoita Creek and Coal Mine Canyon, still exist naturally and at other sites.

Moritz (1994) suggested that reciprocal monophyly for mtDNA sequences be used as a standard for designating ESUs. For Gila topminnows, we found no mtDNA variation within or between these populations, much less reciprocal monophyly. How can we resolve the difference between Moritz’s suggestion and our observation?

First, there has been extensive discussion and criticism on the reliance of reciprocal monophyly of mtDNA as the basis of ESU identity (Waples 1995; Crandall et al. 2000; Fraser and Bernatchez 2001). It is clear that the Gila topminnow Monkey Spring ESU ‘is a product of past evolutionary events and that it represents the reservoir upon which future evolutionary potential depends’ (Waples 1995). Although both Crandall et al. (2000) and Fraser and Bernatchez (2001) suggested alternatives to the ESU approach, it is obvious that the Monkey Spring ESU would qualify as distinctive ‘based on the concepts of ecological and genetic exchangeability’ (Crandall et al. 2000) and would qualify for conservation under the ‘adaptive evolutionary conservation’ scheme envisioned by Fraser and Bernatchez (2001).

Second, Hedrick et al. (2006) showed theoretically that it is unsurprising that no mtDNA variation was observed but extensive nuclear divergence and diversity was found in the US Gila topminnow populations. Using the observed nuclear heterozygosity and genetic distance between populations for the microsatellite loci, they estimated the ancestral nuclear heterozygosity. From this, they predicted the number of generations and effective population size necessary to reduce the heterozygosity as much as observed. Applying this information, and taking into consideration the maternal inheritance and haploidy of mtDNA, only 0.65% of the original mtDNA diversity would be expected to be remaining in the Gila topminnow population. As a result, it does not appear unexpected that there is no mtDNA variation, much less reciprocal monophyly, while there is substantial variation and differentiation for microsatellite and MHC loci. In addition, female topminnows can multiply mate and can store sperm from multiple males, making the potential amount of variation from nuclear genes larger than the four times the expected based on the maternal, haploid inheritance of mtDNA. In fact, a single previously inseminated female could found a new population.

There are not enough data to determine whether different ESUs exist for the Gila or Yaqui topminnow in Mexico although the present data suggest that there may be substantial variation over the distribution of both species. The only available Mexican Gila topminnow data are the two mtDNA sequences from Mateos et al. (2002), somewhat distant in the state of Sonora from the existing US samples, which were about 0.4% different from the US sequence.

All of the Yaqui topminnows from Arizona had identical mtDNA sequences. The two Yaqui topminnow samples from Arizona that have been examined for microsatellite and MHC loci from North Pond and Tule Spring were quite similar, sharing alleles or very similar alleles at all loci (Hedrick et al. 2001a). In other words, these samples could be considered the same ESU and the only known one in the USA. However, the two Yaqui topminnows from Mexico (Mateos et al. 2002), both quite distant from the United States, had sequences that were approximately 0.7% different from the Arizona sequence.

In addition, in a sample of 20 Yaqui topminnows from Cajon Benito, Sonora, Mexico, just south of the border with the United States, and the two examined Arizona Yaqui topminnow populations, eight mtDNA sequences for the three genes (ND2, cytochrome B, and the D loop genes) were found, all different from the Arizona sequence (Hedrick P. W. unpublished data). These sequences differed from the Arizona Yaqui sequence by only one to three nucleotides (of 2626), reflecting the close geographic proximity and evolutionary relatedness of these fish. Further, in this sample of 20 Yaqui topminnows, an additional 19 MHC alleles different from both the 17 Gila topminnow alleles and the previously described 12 Yaqui topminnow alleles were found and 10 new microsatellite alleles were found (Hedrick P.W. unpublished data). In other words, it appears that even nearby Mexican Yaqui topminnow populations harbor substantial variation not found in the US populations and suggests that Yaqui topminnow populations further away may be substantially different.

## Detrimental variation

Perhaps the most important early contribution of genetics to conservation was the recognition of the importance of inbreeding depression, that is, inbreeding resulting in a fitness reduction, an effect thought to be due to increasing the homozygosity of detrimental alleles (Charlesworth and Charlesworth 1999; Charlesworth and Willis 2009). In addition, detrimental mutations with a small selective disadvantage in a small population may become fixed as if they were neutral and the mean population fitness may decline over time (Hedrick 1994; Wang et al. 1999). It is useful to distinguish between these effects (Kirkpatrick and Jarne 2000) and define the genetic load as a reduction in mean fitness of a population compared to a population without lowered fitness from detrimental variation. When populations are crossed and their progeny have a higher fitness than the parents, this is evidence for genetic load within the parental populations and is generally called ‘genetic rescue’ in the crossed population (Tallmon et al. 2004; Hedrick and Fredrickson 2010), or heterosis in plant and animal breeding. When progeny from interpopulation crosses have lowered fitness than the parental populations, this is called ‘outbreeding depression’ (Frankham et al. 2011). Inbreeding depression has been of major concern for endangered species (Hedrick and Kalinowski 2000), and inbreeding avoidance has become a priority in captive breeding.

Documenting the extent of inbreeding depression, genetic load, and outbreeding depression in an endangered species is fundamental to determining its potential for long-term persistence. However, the only other comprehensive examination of these effects in an endangered species to our knowledge, besides our examination discussed below, is a study of inbreeding and outbreeding depression in the oldfield mouse *Peromyscus polionotus phasma* (Brewer et al. 1990). Most of the data examining inbreeding or outbreeding depression in endangered species have been obtained from captive-breeding programs in zoos and other facilities (e.g., Ballou 1997), and in these cases, it is not generally possible to make experimental crosses, maintain simultaneous controls, obtain replicate samples, or even examine more than one component of fitness (generally, only juvenile survival is examined).

Because Gila topminnows can be raised experimentally in captivity, we were able to understand the potential impact of inbreeding and outbreeding depression in them by examining five fitness components or correlates (survival, body size, bilateral asymmetry, brood size, and sex ratio) over two generations of inbreeding (brother–sister mating) or outbreeding in four different populations with simultaneous controls (Sheffer et al. 1999). For example, Table 2 summarizes these results for survival to 12 weeks of age and bilateral asymmetry (relative difference of counts for right versus left) for three traits (pectoral fin rays, pelvic fin rays, and lateral line scales).

**Table 2:** For inbred, outbred, and controls over two generations from four different populations of Gila topminnows, (a) proportion of brood surviving to 12 weeks of age and (b) mean bilateral asymmetry of three traits (pectoral fin rays, pelvic fin rays, and lateral line scales) (Sheffer et al. 1999)

Generation	Population	Inbred	Outbred	Control
(a) Survival				
F_1_	Bylas	1.00	0.85	0.98
	Cienega	0.96	0.98	0.91
	Monkey	0.91	0.94	0.97
	Sharp	0.93	0.85	0.93
	Mean	0.95	0.91	0.95
F_2_	Bylas	0.91	0.92	0.91
	Cienega	0.95	0.94	0.94
	Monkey	–	0.89	0.94
	Sharp	0.94	0.87	0.93
	Mean	0.95	0.91	0.93
(b) Bilateral asymmetry				
F_1_	Bylas	0.00	0.00	0.00
	Cienega	0.12	0.04	0.05
	Monkey	0.04	0.02	0.02
	Sharp	0.02	0.08	0.09
	Mean	0.05	0.03	0.04
F_2_	Bylas	0.00	0.04	0.01
	Cienega	0.08	0.06	0.04
	Monkey	–	0.05	0.10
	Sharp	0.12	0.04	0.19
	Mean	0.07	0.05	0.07

None of these traits values were statistically significant among the inbred, outbred, and control groups after Bonferroni’s correction.

For survival to 12 weeks of age, the rate was quite high, averaging >90% survival over all populations, generations, and types of matings, with the lowest values for Bylas Spring and Sharp Spring outbred matings at 85%. After Bonferroni’s correction, none of comparisons to the control matings were statistically significant. However, note that there were no data for the second-generation inbred category or progeny for Monkey Spring. Similarly, for bilateral asymmetry after Bonferroni’s correction, there were no significant difference between controls and progeny of either inbred or outbred matings. The proportion of completely symmetrical fish for these three traits, pooling over populations and generations, was 0.82 for inbred matings, 0.83 for control matings, and 0.88 for outbred matings.

The only impact of inbreeding found was an extreme one for sex ratio and brood size in the Monkey Spring population. In this cross, only one male was produced (95% females) in the F_1_ inbred progeny and no males in the F_2_ inbred progeny. This female-biased sex ratio under inbreeding may be related to the generation of the parthenogenesis that is found in a number of related Mexican topminnow species (Vrijenhoek 1994). There was no significant impact of outbreeding for any of the traits, consistent with no evidence of either outbreeding depression, genetic load, or genetic rescue (heterosis). As we will discuss below, we did find significant reduction in mating, survival, and brood size in crosses between Gila and Yaqui topminnows.

It is tempting to suggest that these rather low levels of inbreeding and outbreeding depression are associated with the repeated founder events thought to be historically typical of the flood-drought variation often observed in habitats where Gila topminnow populations exist (Minckley 1999). Bottlenecks and/or low effective population sizes may have purged much of the detrimental genetic variation influencing fitness in some of the populations and reducing inbreeding depression. Monkey Spring, the only population that showed significant inbreeding depression is located in a stable warm-water spring and has probably not been subject to this high environmental variation. Overall, the low observed inbreeding depression, except for Monkey Spring, may imply a low amount of standing detrimental variation. Selection among lineages with only those of highest fitness surviving and recolonizing sites during floods may have resulted in reduction in genetic load and lack of outbreeding depression.

## Adaptive variation

The extent and pattern of adaptive (advantageous) variation is crucial to the long-term survival of endangered species. In particular, if there is no standing adaptive variation in a population and it faces a new environmental challenge, such as a new disease or introduced species, its only potential for adaptive response is from new mutations or gene flow from other populations or taxa. However, determining the extent and pattern of adaptive variation in the present or presumed future environments is quite difficult. Potentially, experimental tests of fitness and potential for adaptation in a variety of environments could be carried out, but this is not easy even in a model organism and generally less possible in an endangered species.

### Fitness variation

To examine differential adaptation of populations of Gila topminnows, Sheffer et al. (1997) experimentally examined four fitness correlates: survival, growth rate, fecundity, and bilateral symmetry in four different populations. These experiments were carried out in standardized laboratory conditions in a ‘common garden’ design known for successful topminnow survival and reproduction (25.5°C, 14-h light and 10-h dark, appropriate levels of dissolved gases, solids, and wastes, and a diet of high protein fish food, spinach, and brine shrimp, see Sheffer et al. 1997).

For all the populations, survival was uniformly high, and there was little bilateral asymmetry (80% of the fish were symmetrical for all three traits) (Table 3). For body size (standard length in millimeter at 12 weeks), there was significant variation over populations for both females and males, primarily because the fish from Monkey Spring were larger than the other fish. There was significant variation in brood size in wild-caught fish over populations, with Cienega Creek and Sharp Spring having the largest broods. The brood size for captive-raised fish (the progeny of the wild fish) was much lower and showed no significant variation over populations.

**Table 3:** Survival, mean bilateral asymmetry over three traits, body size for females and males, and brood size, both for wild-caught and for captive-raised fish from four different populations (*P* indicates level of significance, and NS indicates not significant) (Sheffer et al. 1997). All *P* < 0.01 values are significant after a Bonferroni’s correction. Also given is the duration of male development in days (Cardwell et al. 1998)

Trait	Bylas	Cienega	Monkey	Sharp	*P*
Survival	0.90	0.96	0.93	0.96	NS
Bilateral asymmetry	0.09	0.10	0.05	0.05	NS
Size					
Female	26.8	26.5	27.5	26.5	< 0.01
Male	22.3	21.6	24.6	22.7	< 0.01
Brood size					
Wild-caught	12.8	17.8	12.1	15.8	< 0.01
Captive-raised	6.1	6.6	6.0	5.5	NS
Male development	30.4	34.4	47.0	32.7	< 0.01

Earlier research by Quattro and Vrijenhoek (1989) for fish from Monkey Spring and Sharp Spring found only an average 50% survival over populations, 20% smaller fish from Monkey Spring than in Sheffer et al. (1997), and only 22% of the fish from Monkey Spring symmetrical for all three traits. From these results, which showed higher fitness correlate values for Sharp Spring fish, they concluded that Sharp Spring fish were the best choice for stocking new populations. These effects occurred even though the fish were raised at 28°C, higher than the temperature we found appropriate for successful survival and reproduction in experimental laboratory conditions and the same as in constant-temperature Monkey Spring. Sheffer et al. (1997) had more statistical power than did Quattro and Vrijenhoek (1989), that is, the sample size for survival analysis was twice as high as in their experiments. Furthermore, Sheffer et al. (1997) examined four populations, not two, of Gila topminnows (the third population Quattro and Vrijenhoek examined was from the other species, the Yaqui topminnow, which should invalidate its use for interpopulational comparisons).

Although one could suggest that the study of Quattro and Vrijenhoek (1989) provides an examination of fitness correlates in a different and apparently much more stressful environment, the nature of these environmental stressors is not known and they may not even occur in natural environments inhabited by Gila topminnows (see extensive discussion in Sheffer et al. 1997). The eightfold higher mortality in the experiments of Quattro and Vrijenhoek (1989) led R. Vrijenhoek (as quoted by Sheffer et al. 1997) to conclude that ‘obviously our fish were stressed’. Further, he suggested that ‘we have always found that *P. occidentalis* were particularly difficult to maintain under our conditions’, stating that the conditions they used were ‘developed to maximize growth and survival of *P. monacha* and the unisexual (topminnow)…’ and therefore may not be suitable for maintaining Gila topminnows. For Gila topminnows, the major causes of mortality in nature are thought to be predation (including cannibalism), intraspecific density effects, and food limitation (Minckley et al. 1977; Schoenherr 1977). Physicochemical conditions, such as the level of dissolved solids and pH, have not been recognized as major factors in mortality, but they do differ dramatically between the southwestern United States and the northeastern United States where Quattro and Vrijenhoek (1989) carried out their experiments.

To determine the level of bilateral asymmetry found in wild fish, museum samples collected from Monkey Spring in 1955 and Sharp Spring in 1979 (Hedrick unpublished data) and wild-caught fish from both Monkey Spring and Sharp Spring (Sheffer et al. 1998) were examined. Their bilateral asymmetry was similar to that observed in the captive samples in Sheffer et al. (1997) and, for example, 69% of the wild-caught Monkey Spring fish were symmetrical for all three traits (Sheffer et al. 1998), more than three times that observed by Quattro and Vrijenhoek (1989) for Monkey Spring fish.

Observations by Sheffer et al. (1997) suggested that there may be differences in the timing of male sexual maturity among populations, a trait with potentially important fitness consequences. A separate replicated experiment in a controlled and common environment was initiated to measure the rate of male development, specifically the time from the first observation of the male sexual organ, the gonopodium, until completed gonopodial development, signaling male sexual maturity. For this trait, there were highly significant differences, with male Monkey Spring fish having a 45% longer development or a greatly delayed sexual maturity (Table 3). The most obvious explanation for this difference is that the constant warm-water temperature condition of Monkey Spring supports year-round reproduction, while the seasonally variable temperature habitats of the other populations do not. Because of year-round reproduction at Monkey Spring, some females are available for mating all year, which reduces selection against later starting and slower development and consequently larger males. On the other hand, male fish at temperature-variable sites are under selection pressure against late development because reproduction ceases from late fall to spring.

Sheller et al. (2006) evaluated the factors that influenced success in an extensive translocation program for establishing new populations of the Gila topminnow (see also Robinson and Ward 2011). In particular, they found that translocations using stock from Monkey Spring had a population persistence of only 4 years, while translocations using stocks from Bylas, Cienega, or Sharp had a significantly longer persistence of 12 years. Because the habitats of nearly all the translocation sites had seasonally variable temperature and water flow, the lower success of Monkey Spring translocations is consistent with the lower fitness of Monkey Spring fish in these habitats.

### MHC variation

In addition to experimental tests, such as the common garden examination of fitness-related traits just discussed, the extensive molecular data available today potentially provide new ways to determine whether adaptive selection has operated in the past on a given gene and therefore may operate in the future. For example, rather than measuring the impact of selection in a single or a few generations by determining differential viability or reproduction, the cumulative effect over many generations may be observed in the analysis of DNA variation.

Genes in the MHC in vertebrates are involved in pathogen resistance (Hedrick and Kim 2000; Bernatchez and Landry 2003), and molecular variation appears to be important in response to pathogens. One molecular evolution approach that supports this adaptive function for MHC genes is the higher rate of nonsynonymous (amino acid changing) to synonymous substitutions at functionally important amino acid positions (for a summary of tests at MHC, see Garrigan and Hedrick 2003). In the examination of a class II MHC gene in Gila topminnows, Hedrick and Parker (1998) found that 25 of the 29 observed substitutions were nonsynonymous. The estimated rates of nonsynonymous and synonymous amino acid substitutions are given in Table 4 for both Gila and Yaqui topminnows, divided into codons that bind peptides from pathogen molecules to initiate immunological response (peptide-binding sites, or PBS), an important function of MHC molecules, and those that do not. The rate of substitution is higher for PBS codons in both species, and the ratio of nonsynonymous to synonymous substitutions is much higher than 1.0 for PBS sites in both species. These data are consistent with balancing selection operating on this gene in the past and potentially in the present (Garrigan and Hedrick 2003).

**Table 4:** The estimated rates of nonsynonymous (*dN*) and synonymous (*dS*) substitutions for antigen (ABS) and nonantigen binding amino acid sites and their ratio for sequences found in Gila and Yaqui topminnows

Taxa	Position	*N*	*dN*	*dS*	*dN/dS*	*P*
Gila	ABS	20	0.209	0.064	3.27	0.02
	Non-ABS	42	0.045	0.038	1.18	0.40
Yaqui	ABS	20	0.216	0.072	3.00	0.02
	Non-ABS	42	0.079	0.019	4.16	0.01

*N* is the number of codons in each category, and *P* is the probability that *dN* and *dS* are different (data from Hedrick et al. 2001a, b). Using a Bonferroni’s correction for four tests, α = 0.013 so that *P* = 0.01 is significant, while *P* = 0.02 is not.

With the knowledge of MHC variation found in Hedrick and Parker (1998), several experiments were conducted in Gila topminnows to investigate the level of pathogen resistance. For example, Hedrick et al. (2001b) examined the impact of the novel fluke parasite *Gyrodactylus turnbulli* from guppies *Poecilia reticulata* on captive Gila topminnows from four populations. Three general patterns of infection by flukes were observed: resistant hosts where the fluke failed to establish or reproduce, moderately susceptible hosts where the fluke reproduced but the host recovered and eliminated the parasite, and highly susceptible hosts where the parasite grew rapidly and the host died.

**Figure 4:**
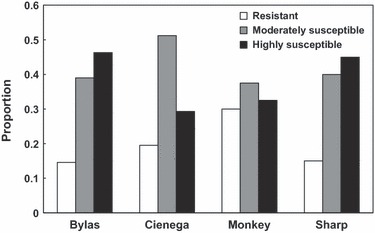
The proportion of Gila topminnows from four populations that were classified as resistant, moderately susceptible, and highly susceptible to infection by an exotic fluke (Hedrick et al. 2001b).

The impact and process of infection can be examined in several ways (Hedrick et al. 2001b), but the overall effect on the three categories of infection for the four populations is given in Fig. 4. Note the Bylas and Sharp Spring fish have the lowest proportion of resistant fish (0.15 in both populations) and the highest proportion of highly susceptible fish (0.46 in Bylas and 0.45 in Sharp). However, because Bylas had the lowest observed heterozygosity for the MHC gene (*H* = 0) (and microsatellite loci) and Sharp Spring had the highest heterozygosity (*H* = 0.60) (and microsatellite loci), there does not appear to be correspondence of MHC heterozygosity and pathogen resistance. When MHC heterozygotes and homozygotes were compared for survival, MHC heterozygotes had higher survival in all three populations, but this difference was not statistically significant (see discussion in Hedrick et al. 2001b).

The impact of the bacterium *Listonella* (formerly *Vibrio*) *anguillarum*, a causative agent of vibriosis in fish, was also examined in the same four populations. The average mortalities from the bacterium for Bylas, Cienega, Monkey, and Sharp were 0.61, 0.69, 0.31, and 0.42, respectively. Although the mortality is again high in the Bylas population with low MHC variation, the mortality for Monkey Spring with relatively low MHC variation (*H* = 0.150) was the lowest. When MHC heterozygotes and homozygotes were compared, the mortality was similar in the three polymorphic populations.

Major histocompatibility complex genes were discovered for their ability to recognize tissue transplants from donors different from the host. Using this ability, pathogen resistance by MHC genes is thought to act through recognition of antigens from bacteria, viruses, and other pathogens. Cardwell et al. (2001), using Gila topminnows, first demonstrated that MHC matching in fish decreased tissue rejection when tissue (scales) were transplanted between individuals, similar to findings in other vertebrates.

Overall, these experiments suggest that there are genetic differences of adaptive significance among these populations. For the novel diseases, they demonstrate MHC variants may not always show significant differences in resistance, and potentially other MHC genes or other genes may provide resistance variation. Positive associations of neutral and adaptive variation may occur when stochastic effects dominate the extent of genetic variation. For example, there is a high association of some microsatellite and MHC loci variation over Gila topminnows (Hedrick et al. 2001a), suggesting that stochastic factors appear important in the present spatial pattern of adaptive variation.

## Reproductive isolation

As we discussed above, based on molecular genetic evidence, the Gila and Yaqui topminnows appear to be species that have been separated for around one million years. In a review of more than 40 pairs of allopatric fish species, McCune and Lovejoy (1998) estimated that the time required for speciation ranges from 0.8 to 2.3 million years. In other words, it is likely that these two taxa should show some extent of reproductive isolation. Attempts previous to our studies to cross these two species were unsuccessful (W. Minckley, personal communication), suggesting that there was reproductive isolation between them. To gain insights into the process of accumulation of biological barriers to reproduction, it is necessary to study species pairs at an incipient stage. The timing of their divergence makes these two endangered species ideal for studying the initial development of reproductive isolating barriers. As a result, because these topminnows can be easily bred and experimentally examined in appropriate laboratory conditions, the system is ideal for the study of speciation processes.

In experimental populations, both premating and postmating reproductive isolation between these two species were examined. First, in no-choice mating trials, there was evidence that mating patterns of the two species had diverged significantly, some evidence of conspecific mate preference, and that Yaqui males appeared to be the more vigorous of the two species (Hurt et al. 2004). In multiple-choice trials (males of both species with females of one species or the other), there was strong evidence of assortative mate preference. In these experiments, males of both species spent more time performing mating behaviors and attempted more copulations toward conspecific than toward heterospecific females. These results are summarized in Table 5, which gives the relative amount of time or activity for four mating behaviors. The assortative preference was asymmetric with Gila topminnows having a stronger preference for conspecific mates than Yaqui topminnows.

**Table 5:** The relative amount of time or activity in four premating behaviors in a multiple-choice experiment (Hurt et al. 2004) and the proportion of successful crosses within and between Gila and Yaqui topminnows, the proportion of female F_1_ progeny, and the days until the F_1_ females produced their first brood (Hurt and Hedrick 2003)

Male	Gila		Yaqui		
			
Female	Gila	Yaqui	Gila	Yaqui	*P*
Premating					
Following	1.0	0.263	0.784	1.0	0.01
Posturing	1.0	0.000	0.611	1.0	0.03
Nibbling	1.0	0.008	0.068	1.0	0.01
Thrusting	1.0	0.051	0.966	1.0	0.04
Postmating					
Successful crosses	0.85	0.54	0.69	0.69	0.04
Female progeny	–	0.08	0.59	–	0.001
Days to first brood	62.4	87.1	53.6	63.7	0.001

To examine postmating reproductive isolation, Hurt and Hedrick (2003) examined reproductive fitness in a series of crosses and backcrosses between the two species. Reciprocal interspecific crosses between species type were made, and both types of interspecific crosses were successful. The lowest success (54%) was for Gila male × Yaqui female cross, a cross that also produced a very skewed sex ratio (only 8% females), and the time to produce the first brood was 45% longer than the average of the other crosses (Table 5).

**Figure 5:**
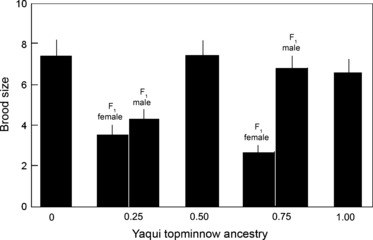
The mean brood size for Gila (indicated by 1) and Yaqui (indicated by 0) topminnows, F_1_ crosses between them (0.5), and the two types of backcross categories (0.25 and 0.75) where these crosses are separated by the sex of F_1_ individuals. Here, the two types of backcrosses in which F_1_ of a given sex were produced by reciprocal matings were combined (Hurt and Hedrick 2003).

To determine whether there was a further reproductive barrier that would inhibit crosses between these F_1_ progeny and the two species, each of the eight types of backcrosses was made. For example, the four crosses that produced 75% Gila topminnow ancestry were Gila males × F_1_ females (from either a Gila male × Yaqui female cross or a Yaqui male × Gila male cross) and F_1_ males (from either a Gila male × Yaqui female cross or a Yaqui male × Gila male cross) × Gila females. High reproductive success was observed across the eight categories of backcrosses with at least 80% of the crosses in each category producing offspring. However, brood size varied greatly by individual cross type. In particular, crosses between F_1_ females and pure species males had a lower average brood size than did the reciprocal cross category (or other categories) (Fig. 5).

Knowledge of the relative contributions of premating and postmating reproductive isolation in taxa that have remained geographically isolated is limited, and the data are not consistent. The most comprehensive analysis has come from the comparative examination of premating and postmating barriers in 171 species pairs of Drosophila (Coyne and Orr (1989, 1997), and these results shower no difference in the genetic distances associated with the premating behavioral and intrinsic postmating barriers in allopatric species pairs. In a similar study of species pairs of the freshwater fish genus *Etheostoma*, Mendelson (2003) found that premating behavioral barriers evolved earlier than postmating barriers.

To evaluate the relative contributions of the premating and postmating reproductive isolation that we documented between Gila and Yaqui topminnows, we established 22 replicate populations initiated with equal numbers of virgin males and females of both species (Hurt et al. 2005). Using mtDNA, MHC, and microsatellite markers, we monitored these populations for two generations and predicted the change in ancestry from the two taxa using populations genetic theory and assortative mating estimates from Hurt et al. (2004) and fitness values from the reproductive experiments of Hurt and Hedrick (2003).

**Figure 6:**
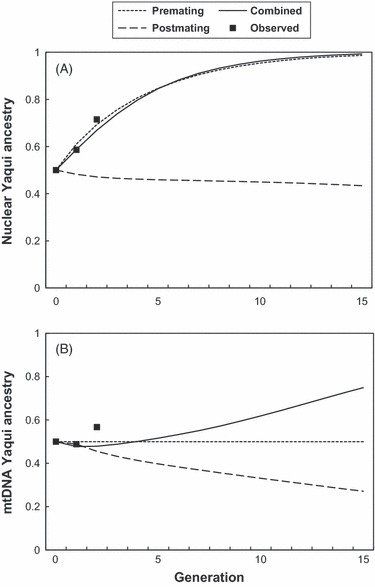
The change in the frequency of Yaqui topminnow ancestry over time for (A) nuclear genes and (B) mtDNA. The closed circles indicate the average observed frequencies, and the predicted changes with only premating, only postmating, and combined premating and postmating are indicated by the short broken line, long broken line, and solid line, respectively (Hurt et al. 2005).

Figure 6 gives the expected change in the proportion of Yaqui topminnow ancestry for nuclear and mtDNA genes when there is only premating, only postmating, or combined premating and postmating reproductive isolation. The expected pattern for premating isolation was that Yaqui nuclear ancestry would quickly dominate and Yaqui mtDNA ancestry would not change, while the prediction for postmating isolation was that there would be little change from the initial ancestry for nuclear genes and a slow decline for mtDNA genes. When both premating and postmating isolations were combined, the predicted results were nearly identical to that for premating isolation for nuclear genes, but for mtDNA genes, the Yaqui ancestry increased.

In our experimental populations, the Yaqui nuclear ancestry increased in both generations and the Yaqui mtDNA ancestry increased in generation 2. In other words, these observations were consistent with the predictions from the premating and combined models for nuclear genes and consistent with the combined model for mtDNA genes. Overall, these results suggest that, given the amount of premating and postmating isolation operating and estimated in these two species, premating barriers would drive the genetic dynamics in hybrid populations. As for conservation implications, one would predict from these findings that if Gila and Yaqui topminnows were not kept apart, the Yaqui topminnow advantage would drive Gila topminnow ancestry to extinction.

## Conclusions

Since conservation biology became a separate discipline in the 1980s, there have been efforts to establish broad principles for use over different threatened and endangered species. In particular, conservation genetics has developed significantly resulting in several comprehensive textbooks (Allendorf and Luikart 2007; Frankham et al. 2010) that generalize conservation assumptions and guidelines. However, most endangered species have some characteristics that set them apart from other related species, and it seems unlikely that examination of model organisms or more common organisms contain all aspects of an endangered species. As a result, the research we have discussed here presents an extraordinary examination of many evolutionary and genetic aspects of a highly endangered species, a situation not comparable in any other endangered species.

As we have demonstrated above, the Gila and Yaqui topminnows can be used to investigate experimentally many of the factors thought important in its endangerment. In particular, neutral genetic variation has been used to determine both the presence of species and ESUs. Two generations of inbreeding and outbreeding in the laboratory have been used to characterize detrimental genetic variation within and between populations. Adaptive variation has been documented by examination of fitness-related differences over populations, variation in an MHC gene, and the association of MHC variation with resistance to novel pathogens. Finally, experimental determination of incipient premating and postmating reproductive isolation between Gila and Yaqui topminnows has been experimentally documented, and the impact of this reproductive isolation over time is examined both experimentally and theoretically. In other words, fundamental biological questions have been examined experimentally in these endangered species providing important background for understanding the evolutionary history of the species and their potential recovery.

Can these findings be generalized to other endangered species? For other species that have a similar history of population contraction and expansion, the observations related to the lack of pattern of mtDNA over ESUs may provide an important lesson. Also, low levels of inbreeding and outbreeding depression (and genetic load) may also be found in species with similar population dynamics. For example, desert bighorn sheep now exist in small, isolated mountain populations and may have similar patterns of inbreeding and outbreeding depression to those in topminnows. In spite of these findings, the pattern of adaptive differences over populations, particularly the differences seen between the constant environment of Monkey Spring and the other habitats, demonstrate that selective forces can be important even in a species apparently dominated by stochastic forces. In other words, some of these conclusions may be fairly specific to these species but may be shared or important in species with similar attributes.

When the studies discussed above were carried out, mainly between 1995 and 2005, there was funding for study of endangered species and these species in particular. Now, there is little funding for basic conservation research from federal agencies or state resources. As a result, it is unlikely that a thorough examination of many aspects of conservation genetics of endangered species, like that undertaken for the Gila and Yaqui topminnows discussed here, could be carried out at this point. One of our goals here is to show that genetics and evolution studies in endangered species can provide important insights into conservation and recovery, and it is hoped that this may generate support for future research in conservation genetics.
